# Living in a high-tech world yet dragging an ancient problem: water insecurity

**DOI:** 10.1016/j.lana.2024.100951

**Published:** 2024-11-12

**Authors:** The Lancet Regional Health – Americas

While the world continues to accelerate the development of highly sophisticated and needed technologies (nanochips, zero-emission cars, artificial intelligence, and monoclonal antibodies, to name a few), we have been unable to solve one of the most pressuring problems in public health: permanent access to safe water, a fundamental human right. Water demand continues to increase as the global population grows, leading to water insecurity—lack of access to safe water or insufficient water supply. Water insecurity affects people from countries of all income levels. While the quality of drinkable water is the biggest issue in some regions (e.g., Fresno, CA, USA), water scarcity is the biggest problem in other areas, both rural (e.g., the Province of Chaco, Argentina) and suburban (e.g., the Castilla district, Piura, Peru). Water insecurity is not a new problem. Poor water sanitation linked to a deadly cholera outbreak in London, UK, in 1854, gave birth to modern Epidemiology. However, concerns about water sanitation and management actually go back to ancient civilizations.

According to the World Bank, 150 million people live in highly water-scarce areas in Latin America. National surveys suggest that water insecurity experience (self-reported data) could be as high as 48% in Peru and 47% in Honduras. In communities across the region with access to water, the quality might be suboptimal or even unhealthy because of unsupervised sanitation of water sources, for instance, due to highly salinized water or contamination with heavy metals. The consequences of water insecurity on people’s health include poor sanitation; metal and radioactivity exposure; and waterborne diseases, which could also lead to acute respiratory diseases and undernutrition, dehydration, heat stroke, and even death. It has been estimated that 1.4 million deaths globally could have been prevented in 2019 by safe water, sanitation, and hygiene (WASH) practices.

It took 62 years for the UN to declare safe and clean water as a human right after the first official international declaration of human rights was published in 1948. Since the latest declaration by the UN in 2010, the worrying statistics have improved slightly. Between 2015 and 2022, the proportion of the world’s population with access to safe water increased from 69% to 73%. However, little is known if this is a global trend or if the numbers are driven by data from a few countries. Nevertheless, these statistics show that we are still far from universal water security. Declaring safe water access a human right and setting ambitious goals like the UN Sustainable Development Goal 6 are big steps. However, to revert the water crisis, we require more planning and strategies, more international cooperation, and more rational use of water for agriculture, which currently uses 70% of the global freshwater. Overall, the largest external use of water occurs in irrigation projects and hydroelectric power plants. We also need to use water more wisely in our daily lives. For example, in the USA, the average family consumes nearly 10,000 gallons of water per month, of which 30% is used outdoors.

To tackle water crisis, we need more commitment from country, regional, and local governments, and also zero corruption, as this can affect public expenditure on essential projects and hinder economic growth. This task is going to be even more difficult in the coming years as the problem will worsen due to climate change, which causes droughts and floods, disrupting water supply and safe water. For instance, in west USA, water insecurity has been linked to drought, which is becoming more common due to climate change.

WHO has pointed out the risks associated with unsafe water and produced comprehensive guidelines. But the bottleneck is in the implementation of policies by governments, which has to do with political will. Equally important is the actual monitoring of water service, the development of more innovative ways to redistribute water, and anticipation of service problems to prevent prolonged water disruption. Progress has certainly been made, such as the Norte Grande Water Infrastructure Project that provided safe water access to more than 50 000 people in El Chaco, Argentina. This type of intervention, focused on improving infrastructure, should be massively implemented by governments as a priority in those communities that have no access to drinkable water.

To tackle water insecurity, we must consider all the factors associated with it, such as population growth and consequent high water demand, industrialisation (including agriculture), poor awareness of water conservation, and climate change. Water insecurity is also linked to poverty. For instance, people with higher incomes in Lima, Peru, who have access to safe water, pay an average of US$0.87 per cubic meter of water. Ironically, people living in the areas with the highest poverty levels in the city, who depend on water trucks, must pay much more: from $3.9 to $10.4 per cubic meter, depending on the elevation at which their homes are located in the inaccessible bald and arid hills.

Along with policy implementation and monitoring, more research is needed to inform policy makers to make wise decisions in a world with so many competing needs. Further research is also urgently needed to better understand the actual status of water insecurity across countries in the Americas, and worldwide, and its burden on people’s health and economies.

*The Lancet Regional Health—Americas* welcomes epidemiological studies on water crisis in the region and game-changer health policy recommendations to address this major issue in public health.

